# Antarctic Extremophiles: Biotechnological Alternative to Crop Productivity in Saline Soils

**DOI:** 10.3389/fbioe.2019.00022

**Published:** 2019-02-19

**Authors:** Ian S. Acuña-Rodríguez, Hermann Hansen, Jorge Gallardo-Cerda, Cristian Atala, Marco A. Molina-Montenegro

**Affiliations:** ^1^Center for Molecular and Functional Ecology (CEMFE), Instituto de Ciencias Biológicas, Universidad de Talca, Talca, Chile; ^2^Facultad de Ciencias Agrarias, Universidad de Talca, Talca, Chile; ^3^Laboratorio de Anatomía y Ecología Funcional de Plantas (AEF), Instituto de Biología, Pontificia Universidad Católica de Valparaíso, Valparaiso, Chile; ^4^Centro de Estudios Avanzados en Zonas Áridas (CEAZA), Facultad de Ciencias del Mar, Universidad Católica del Norte, Coquimbo, Chile

**Keywords:** extremophiles, Antarctica, functional symbiosis, crops, food security, salt tolerance

## Abstract

Salinization of soils is one of the main sources of soil degradation worldwide, particularly in arid and semiarid ecosystems. High salinity results in osmotic stress and it can negatively impact plant grow and survival. Some plant species, however, can tolerate salinity by accumulating osmolytes like proline and maintaining low Na^+^ concentrations inside the cells. Another mechanism of saline stress tolerance is the association with symbiotic microorganism, an alternative that can be used as a biotechnological tool in susceptible crops. From the immense diversity of plant symbionts, those found in extreme environments such as Antarctica seems to be the ones with most potential since they (and their host) evolved in harsh and stressful conditions. We evaluated the effect of the inoculation with a consortium of plant growth-promoting rhizobacteria (PGPB) and endosymbiotic fungi isolated from an Antarctic plant on saline stress tolerance in different crops. To test this we established 4 treatments: (i) uninoculated plants with no saline stress, (ii) uninoculated plants subjected to saline stress (200 mM NaCl), (iii) plants inoculated with the microorganism consortium with no saline stress, and (iv) inoculated plants subjected to saline stress. First, we assessed the effect of symbiont consortium on survival of four different crops (cayenne, lettuce, onion, and tomato) in order to obtain a more generalized response of this biological interaction. Second, in order to deeply the mechanisms involved in salt tolerance, in lettuce plants we measured the ecophysiological performance (F_v_/F_m_) and lipid peroxidation to estimate the impact of saline stress on plants. We also measured proline accumulation and *NHX1* antiporter gene expression (involved in Na^+^ detoxification) to search for possible mechanism of stress tolerance. Additionally, root, shoot, and total biomass was also obtained as an indicator of productivity. Overall, plants inoculated with microorganisms from Antarctica increased the fitness related traits in several crops. In fact, three of four crops selected to assess the general response increased its survival under salt conditions compared with those uninoculated plants. On the other hand, saline stress negatively impacted all measured trait, but inoculated plants were significantly less affected. In control osmotic conditions, there were no differences in proline accumulation and lipid peroxidation between inoculation treatments. Interestingly, even in control salinity, F_v_/F_m_ was higher in inoculated plants after 30 and 60 days. Under osmotic stress, F_v_/F_m_, proline accumulation and *NHX1* expression was significantly higher and lipid peroxidation lower in inoculated plants compared to uninoculated individuals. Moreover, inoculated plants exposed to saline stress had a similar final biomass (whole plant) compared to individuals under no stress. We conclude that Antarctic extremophiles can effectively reduce the physiological impact of saline stress in a salt-susceptible crops and also highlight extreme environments such as Antarctica as a key source of microorganism with high biotechnological potential.

## Introduction

As a result of the global demand for processed food, intensive agricultural practices had altered the natural dynamics of the soil around the world, leading in many cases to the degradation of edaphic properties that are fundamental for crop productivity (Godfray et al., 2010). Rainfall and evapotranspiration dynamics, like those experienced by soils from arid and semiarid regions, could naturally produce a high concentration of diluted salts in the edaphic plant-rooting zone (Zhu, 2001; Zheng et al., 2009). However, several key aspects of intensive agricultural systems, like assisted irrigation, are actually one of the main drivers of soil saline accumulation (Allbed and Kumar, 2013). This process, known as soil salinization, represents one of the major forms of land degradation and was proposed to be a global problem for food security in the upcoming decades (Food and Agriculture Organization of the United Nations, 2009).

Today, agricultural productivity of arid and semiarid regions has become the most affected by salinity around the world (Rozema and Flowers, 2008), and traditional agricultural practices are already being challenged to maintain crop productivity. The pressure on agricultural soils will likely increase at a global scale in the future. This mainly due to the increased food demand as the world's population increases (Godfray et al., 2010), and the expected restrictions in water use as predicted by climate change models. Thus, alternative practices that allow the utilization of degraded soils for crop production could became necessary in the near future. One of these practices, plant-microbe symbiosis, is highlighted as one of the most promising tools in such context (Paul and Lade, 2014; Joshi et al., 2015). For this reason, the mechanistic analysis of this kind of interactions, and their evaluation for crop production, must be of primary interest in the upcoming years.

Whereas a consequence of low osmotic potential (Munns and James, 2003), specific saline ion stress (Tester and Davenport, 2003) or nutritional imbalances derived from saline edaphic accumulation (Ashraf and Harris, 2004), salinity on soils can cause strong adverse effects on plant growth and development in several crop species (Glenn et al., 1999). Nevertheless, plants have developed a wide variety of mechanisms to cope with high saline concentrations in the environment (Munns, 1993; Forni et al., 2017). For example, at the cellular scale, the accumulation of organic osmolytes (e.g., proline, glycine, betaine, sugar alcohols, or polyamines), play a key role in preventing the harmful effects of salinity stress by maintaining a low intracellular osmotic potential (Pandey et al., 2015; Reddy et al., 2015; Saha et al., 2015). This was clearly demonstrated in the *Arabidopsis thaliana* knockout variety P5CS1, which fails to encode Δ-1-pyrroline-5-carboxylate synthetase, impairing proline synthesis, which resulted in a salt hypersensitive plant (Székely et al., 2008; Szabados and Savoure, 2010). Other mechanisms of saline-stress control are related to the maintenance of low cytoplasmic Na^+^ concentrations. This could be achieved by the augmentation of the tonoplast-localized Na^+^/H^+^ exchanger protein NHX1, which detoxifies the cells via sequestration of Na^+^ cations within the vacuole (Sun et al., 2017). In this sense, strategies at the genetic scale, as the constitutive over-expression of the *AtNHX1* gene have shown increases on plant tolerance to salinity in *Arabidopsis* sp., but also in important crops like tomato and rice (Assaha et al., 2017; Sun et al., 2017).

Despite significant efforts (Munns and Tester, 2008; Dodd and Pérez-Alfocea, 2012; Coleman-Derr and Tringe, 2014; Krishna et al., 2015), traditional breeding and genetic engineering approaches have had only limited successes in developing salinity-resistant plants. However, the utilization of microbial symbionts has arisen as a successful (and relatively simple) biotechnological tool to achieve this goal (Bianco and Defez, 2009; Karlidag et al., 2013; Kang et al., 2014; Radhakrishnan et al., 2015; Forni et al., 2017). In fact, due to their active role in the regulation of a wide variety of plant physiological responses, the inoculation of specific microbial species on different plant hosts has been linked to enhance stress-tolerance responses against both biotic and abiotic factors (Cocking, 2003; Redman et al., 2011; Kang et al., 2015; Rho et al., 2017). However, despite the wide diversity of microbial symbionts that are present on almost all plant species; those associated with plant hosts from extreme environments appear to be the most promising in developing biological alternatives to assist crop production (Molina-Montenegro et al., 2016; Orhan, 2016; Qin et al., 2016). This could be consequence, at least in part, of the adaptive nature supposed for asymptomatic plant-microbe interaction in extreme environments (Saikkonen et al., 1998); but also because the selective pressure that these kinds of ecosystems imposes on plants demands specific biological responses, in this case, via symbiotic interactions (Redman et al., 2011).

Consequently, the Antarctic terrestrial ecosystem appears as a promising place to find plant-associated microorganisms that could confer plants protection against abiotic stress (Upson et al., 2009; Santiago et al., 2017; Gallardo-Cerda et al., 2018; Ramos et al., 2018). Besides its isolation, Antarctica is characterized by harsh environmental conditions like low temperatures, low water content, low nutrient availability and saline soils (Convey et al., 2008; Gallardo-Cerda et al., 2018). In such conditions, only two native vascular plants grow: *Colobanthus quitensis* and *Deschampsia antarctica* (Alberdi et al., 2002). In the last 10 years, the interest in microorganisms associated to these vascular plants has greatly increased, finding promising bacterial and fungal strains with a wide spectrum of applications (Parnikoza et al., 2011; Fardella et al., 2014; Molina-Montenegro et al., 2016; Santiago et al., 2017).

Related to the saline stress tolerance, the microbial symbionts of both Antarctic vascular plant species appeared as great candidates to explore this role, since at the local scale these plants can be found primarily along the ice-free zones close to the coast, where their populations are constantly subjected to saline spray influence (Convey et al., 2008). For example, Gallardo-Cerda et al. (2018) showed the beneficial effect of various Antarctic rhizospheric bacteria (e.g., *Arthrobacter* sp.) in the physiology and survival of both native plants under controlled conditions of salt stress (200 mM of NaCl). Similarly, Torres-Díaz et al. (2016) found a strong functional role of the microbial symbionts of *C. quitensis*, particularly its root-fungal endophytes, in the ecophysiological improvement of plant individuals under osmotic stress. Interestingly, some of these Antarctic microorganisms (i.e., root-fungal endophytes and halotolerant rhizobacteria) have also shown the potential to improve the physiological performance of non-native host plants (Fardella et al., 2014), which could be quite important for a future use as biotechnological tool. Nevertheless, beyond the positive effects observed in some crops as a consequence of a manipulative symbiosis, there is still a lack of understanding regarding the mechanisms behind these positive effects under stressful conditions. For example, for most crop-microbial symbiosis, it is not clear if the observed benefits are a direct consequence of counteracting specific abiotic stressors (i.e., increased tolerance; Kim et al., 2014; López-Gómez et al., 2014), or an indirect effect derived, for example, from an enhanced nutritional status (Upson et al., 2009; Dodd and Pérez-Alfocea, 2012). On the other hand, some studies have been conducted to test the effect of microorganisms associated to Antarctic plants on stress tolerance in others plants, but all of them have been addressed using only an specific functional group (i.e., rhizobacteria or fungal), but neither has assessed the “consortium.” This is surprising, because in a realistic scenario these functional groups are interacting in a determined way, exerting an inhibitory, neutral or synergic effect among then. In fact, the effect of isolated specific functional group can exert a different effect compared when is considered as part of the microbiome (Vandenkoornhuyse et al., 2015). In this sense, to determine the biotechnological potential of a given crop-microbe functional symbiosis against a particular stressor like saline soils, it is important to identify which response mechanisms are triggered in the host plant as a consequence of the microbial presence as well as to determinate the effects of functional groups acting as part of the microbiome of a given plant species rather than as isolated symbionts.

Hence, to evaluate the potential of a selected group of Antarctic extremophiles to ameliorate the stressful effect of soil salinization on susceptible crops, we monitored the physiological (individual) and biochemical (cellular) performances of inoculated and non-inoculated lettuce plants exposed for 60 days to saline stress. Furthermore, to determine the potential mechanisms involved in the observed stress-response enhancement, we analyzed the foliar accumulation of proline and the relative expression of saline-stress related genes like *NHX1*. Finally, we established the overall effect of the interaction between saline stress and symbiosis on the final biomass of lettuce individuals as an indicator of crop productivity.

## Materials and Methods

### Generation of the Symbiont Consortium

The consortium of microorganisms used in our study was composed by two halotolerant plant growth-promoting rhizobacteria (PGPR) of the genus *Arthrobacter* sp. and *Planoccocus* sp. and two root-fungal endophytes; identifies as *Penicillium chrysogenum* and *Penicillium brevicompactum*. In brief, both rhizobacteria used for this study were selected because are one of the most abundant in the rhizosphere of antarctic plants, ease to cultivate and has been demonstrated to growth under high salt concentrations (Gallardo-Cerda et al., 2018). In addition, these rhizobacteria has been showed to improve the ecophysiological performance of their native hosts in Antarctica (Gallardo-Cerda et al., 2018). On the other hand, root fungal endophytes selected to use in this study were selected because are the two most abundant in the root of Antarctic plants and can be grown at different temperatures and abiotic conditions in the laboratory (Molina-Montenegro et al., 2016). In addition, these endophytes have been demonstrated to improve the ecophysiological performance and productivity in crops under osmotic stress (Molina-Montenegro et al., 2016). Both rhizobacteria and fungal endophytes were identifies and its sequences deposited in the gene-bank. These inoculums are maintained as part of the collection of microorganisms of the Plant Ecology Laboratory, Universidad de Talca, Chile. The inoculums were separated in different Petri dishes and then frozen until to be used in the experiments.

For more details of isolation, inoculation, and the gene-bank code (see Molina-Montenegro et al., 2016; Gallardo-Cerda et al., 2018).

Plant inoculation was performed according to the procedure describe by Hadi et al. (2010) with the selected consortium of microorganisms. Briefly, the rhizosphere of each crop individual was injected with ~2 ml of distilled water containing a concentrated mix of spores (5,000 spores ml^−1^) from each fungal endophyte, and about 10^8^ cells ml^−1^ of the bacterial species. A posterior validation of the effectiveness of the inoculation was conducted using a light microscope in a subsample of roots (10% of the experimental individuals) 1 week after inoculation, and at the end of the experiment (60 days).

Fresh inoculums were obtained during March 2017 from single-conidia of fungal endophytes cultured on potato dextrose agar (PDA) medium diluted eight times and supplemented with 50 mg/ml of streptomycin. Cultures with endophytes were incubated at 22 ± 2°C with a photoperiod 14/10 day/night. After 2 weeks of incubation, conidia were harvested from plates by adding 10 ml of sterile water and gently scraping off conidia with a sterile glass slide. The conidia suspension was adjusted to 100 ml of 0.05% Tween-100, sterilized solution, filtered through three layers of sterile cotton cheesecloth gauze. Conidia concentration was estimated by using a Neubauer chamber and adjusted to 1 × 10^5^ conidia/ml and its viability was tested according to methodology described by Greenfield et al. (2016) and the mean conidia viability was >95%. In addition, rhizobacteria used in this assays were cultured in Laura Bertani broth (LB). Later, they were mixed on an orbital shaker with a speed of 120 rpm and incubated at 10°C for 72 h. The incubated broth cultures were then centrifuged for 15 min at 3,000 rpm. Pelleted cells were suspended in sterile distilled water and its optical density was adjusted to about 10^8^ cells ^*^ml^−1^. Symbiont consortium (fungal endophytes + rhizobacteria) was injected in the rhizosphere, according the procedure describe by Hadi et al. (2010).

### Overall Assessment of Symbiont Consortium in Crops

To test the overall effect of symbiont consortium on salt tolerance in crops, we compared the survival percentage of individuals of cayenne (*Capsicum annuum*), lettuce (*Lactuca sativa*), onion (*Allium cepa*), and tomato (*Solanum lycopersicum*) inoculated and un-inoculated with microorganisms as well as exposed to salt-stressed and control condition. The plant inoculation was repeated two times to ensure the consortium to establish an effective association. Before the beginning of the experiment, three plants of each species/treatment were sacrificed to check microscopically for the presence and/or absence of microorganisms by routine staining of roots.

Seedlings of these crop species were obtained from seeds germinated in glasshouse located at the Universidad de Talca, Talca, Chile (35.4°S), under semi-controlled environmental conditions of light and temperature (730 ± 77 μmol m^−2^s^−1^; 23 ± 5°C, respectively). For treatment setup, crop seedlings were transplanted into the field when individuals presented at least four expanded leaves and/or 3-cm roots. One-hundred seedlings of each species were randomly assigned to one of the following treatments: (i) *Saline stress*: control individuals watered without salt addition, and stressed individuals watered with a 0.2 M saline water solution; (ii) *Symbiont inoculation*: control plants (S–) without the set of Antarctic extremophile microorganisms, and infected plants (S+) which were inoculated with the symbiont consortium described above. Thus, the four treatments corresponded to S+ plants with and without salt stress and S– plants with and without salt stress, with a sample size of 25 plants per treatment per crop species (total *n* = 400 individuals). Finally, each group of 25 individuals was grouped in five group with five individuals each one. Survival percentage was recorded at 60 days on each group of five seedlings per treatment in the field.

### Experimental Arrangement to Evaluate Salt Tolerance in Crops

We decided to use lettuce as a model crop to deeply the understanding about mechanisms and consequences of microorganisms to confer environmental tolerance in crops. This crop has been shown to be highly sensitive and dependent on water at all developmental stages (Sánchez, 2000; Molina-Montenegro et al., 2011), and demands constant watering to maintain high photosynthetic rates and a fresh biomass of high commercial value (Nissen and San martín, 2004). Complementarily, lettuce has also shown the ability to accept non-native microbial symbionts and moreover, to respond positively to these new partners (Molina-Montenegro et al., 2016). Consequently, during April 2017, 500 lettuce seeds (var. Romaine) were germinated in greenhouse conditions. After 3 weeks, 125 seedlings were transplanted to individual pots containing 500 ml of a peat: perlite: sand mix (35%: 35%: 30%) as substrate. Their successful establishment was verified for 1 more week previous to any experimental treatment.

From the set of 114 successfully established seedlings, 100 individuals were selected for the experiments and were then randomly divided into four groups of 25 to fulfill the factorial combination of the two experimental variables of saline stress and effective symbiosis (2 × 2 full factorial design). The respective treatments were (i) *Saline stress*: control individuals watered without salt addition, and stressed individuals watered with a 0.2 M saline water solution; (ii) *Symbiont inoculation*: control plants (S–) without the set of Antarctic extremophile microorganisms, and infected plants (S+) which were inoculated with the symbiont consortium. Thus, the four treatments corresponded to S+ plants with and without salt stress and S– plants with and without salt stress, with a sample size of 25 plants per treatment. The referred consortium of microorganisms was the same to those used to assess the overall effect on survival of several crops indicated above. Similarly, a posterior validation of the effectiveness of the inoculation was conducted using a light microscope in a subsample of roots (10% of the experimental individuals) 1 week after inoculation, and at the end of the experiment (60 days).

### Estimation of the Saline Stress Effect

Having validated the symbiosis by re-inoculation and visual assessment in root samples of experimental plants, half of the 4-weeks old inoculated seedlings, started their saline-stress treatment included in the watering routine. We estimated the photochemical efficiency and the membrane lipid peroxidation state as two physiological/biochemical proxies of osmotic stress (Egert and Tevini, 2002; Molina-Montenegro et al., 2013). To measure chlorophyll fluorescence we obtain the minimum (*F*_0_) and maximum (*F*_m_) fluorescence yields per individual (*n* = 10) using a pulse modulated-amplitude fluorimeter (FMS 2, Hansatech, Instrument Ltd, and Norfolk, UK). The baseline (*t*_0_) measurements were taken previous to the saline addition and were also estimated after 30 (*t*_30_) and 60 (*t*_60_) days. In each case we calculated the maximum quantum yield of photosystem-II (*F*_v_/*F*_m_), where *F*_v_ refers to *F*_0_ – *F*_m_ (Maxwell and Johnson, 2000). To ensure maximum photochemical efficiency, the chosen leaves from each individual were dark-adapted for 30 min previous to the measurements.

Complementary to the individual level of response denoted by the photochemical efficiency, we estimated the state of lipid oxidative degradation by the thiobarbituric acid reactive substances (TBARS) assay (Egert and Tevini, 2002), which measures the concentration of malondialdehyde (MDA) at the cellular level as a proxy of cell damage. For each experimental group, the lipid peroxidation state in 0.5 g of fresh foliar tissue samples from five random individuals was estimated at each time (i.e., *t*_0_, *t*_30_, and *t*_60_). Samples were prepared and homogenized with 2 ml of TCA (1%) to be later centrifuged at 10,000 G for 5 min. For each sample, the supernatant was mixed with 1 ml of 0.5% thiobarbituric acid in TCA (20%) and then incubated for 30 min in boiling water. Once cooled to room temperature, the absorbance of each sample was determined at 532 nm and non-specific absorbance at 600 nm (Hodges et al., 1999). The MDA content was determined by its molar extinction coefficient of 155 mM^−1^ cm^−1^.

### Mechanisms of Saline-Stress Tolerance

To determine the proline accumulation in the foliar tissues of the experimental plants we followed most of the Bate's method (Bates et al., 1973). Samples of fresh healthy leaves (~250 mg) were frozen at each time (i.e., *t*_0_, *t*_30_, and *t*_60_) from the same individuals selected for the TBARS assay. From each individual, a sample of 100 mg was grinded in 1.2 ml of 3% sulfosalicylic acid. The resulting homogenate was then centrifuged at 16,000 G for 20 min to obtain 1 ml of the supernatant, which was added to 2 ml of ninhydrin reagent (2.5% ninhydrin in glacial acetic acid: distilled water: 85% orthophosphoric acid [6:3:1]). Each reaction mix was kept for 1 h in water bath at 90°C to develop color. After cooling the test tubes in an ice-bath, 2 ml of toluene was added to separate the chromophores in the sample. Finally, the toluene phase absorbance was read in a spectrophotometer at 525 nm to further estimate proline concentration by comparing sample absorbances with the standard proline curve.

In addition to the biochemical mechanisms mentioned above, genetic responses of lettuce to saline stress were assessed at each time (i.e., *t*_0_, *t*_30_, and *t*_60_) through the expression of the *NHX1* antiporter gene among a subsample of individuals (*n* = 5) from each experimental group. To achieve this, total RNA was extracted from foliar samples according to Chang et al. (1993). DNA was removed from aliquots of total RNA using TURBO DNA-free (Applied Biosystems, USA). Synthesis of the cDNA strand was performed according to Ruíz-Carrasco et al. (2011). The quantitative PCR (qPCR) reaction contained the cDNA, 5 *p*mol of each primer, and 12.5 ml of the Fast SYBR Green PCR master mix (Applied Biosystems. USA). The sequences used to amplify *LsNHX1* amplicons (~200 bp) were: 5′-GCACTTCTGTTGCTGTGAGTTCCA-3′ (forward); 5′-TGTGCCCTGACCTCGTAAACTGAT-3′ (reverse). PCRs were performed on a Step-One Plus 7500 thermocycler (Applied Biosystems, USA). The process includes an initial cycle of 30 min at 45°C and 2 min at 95°C, and then 40 cycles as follows: 95°C for 30 s, 60°C for 30 s, 72°C for 2 min, and finally one cycle at 72°C for 10 min. Cycle threshold (Ct) values were obtained and analyzed with the 2^−ΔΔCT^ method (Livak and Schmittgen, 2001). The Elongation Factor 1a (EF1a) housekeeping gene was used as reference gene to normalize, and estimate up- or down-regulation of the target genes for all qPCR analyses: 5′-GTACGCATGGGTGCTTGACAAACTC-3′ (forward); 5′-ATCAGCCTGGGAGGTACCAGTAAT-3′ (reverse). The relative expression ratio (log_2_) between the target gene and EF1a, as well as the fold changes (FC) between controls plants (non-inoculated and without salt stress) and experimentally manipulated individuals were calculated from the qRT-PCR efficiencies and the crossing point deviation using the mathematical model proposed by Pfaffl (2001).

### Symbiont Consortium on Crops Productivity

As a commercial crop, the individual final yield of the monitored lettuce plants was averaged in each experimental group. At the end of the experiment (day 60), all remaining lettuces in the experiment were carefully harvested and their roots gently washed with tap water to separate above (leaves) and belowground (roots) tissues in each individual. After 1 h of drying in the shade, their respective fresh weights were obtained using a digital scale (Boeco BBL-52; 0.01 g-precision). Finally, total dry biomass was measured after whole lettuce individuals were over-dried at 62°C for 96 h and the constant weight of biomass was achieved.

### Statistical Analysis

To evaluate the effect of symbiont consortium on the average survival percentage in different crops was used a two-way ANOVA with inoculation and salt as independent variables. The effect of microbial inoculation and saline stress on lettuce plants traits was assessed by repeated measures (rm) ANOVAs on the following variables: proline accumulation, lipid peroxidation (TBARS), *NHX1* relative gene expression, and photochemical efficiency (F_v_/F_m_). Non-parametric mixed model fitting was performed with the *lme* function on the *nlme* R-package using the individual nested in time as the random error structure (Pinheiro et al., 2017). *A posteriori* comparisons for the mixed models between experimental groups were performed by the comparison of their Estimated Marginal Means (EMMs), as supported by the pair function in the *emmeans* R-package (Lenth et al., 2018). Differences in final fresh biomass were analyzed by independent two-way standard ANOVAs for each kind of tissue (root, shoot or the whole plant). We used the presence of the microbial symbionts (inoculated: S+ or non-inoculated: S–) and stress status (no stress: control; saline-stress: stressed) as explanatory variables. When significant results were obtained, *a posteriori* differences between experimental groups were analyzed by the Honest Significant Differences (HSD) Tukey test (Rohlf and Sokal, 1981).

## Results

### Overall Assessment of Symbiont Consortium in Crops

**Figure 1:**
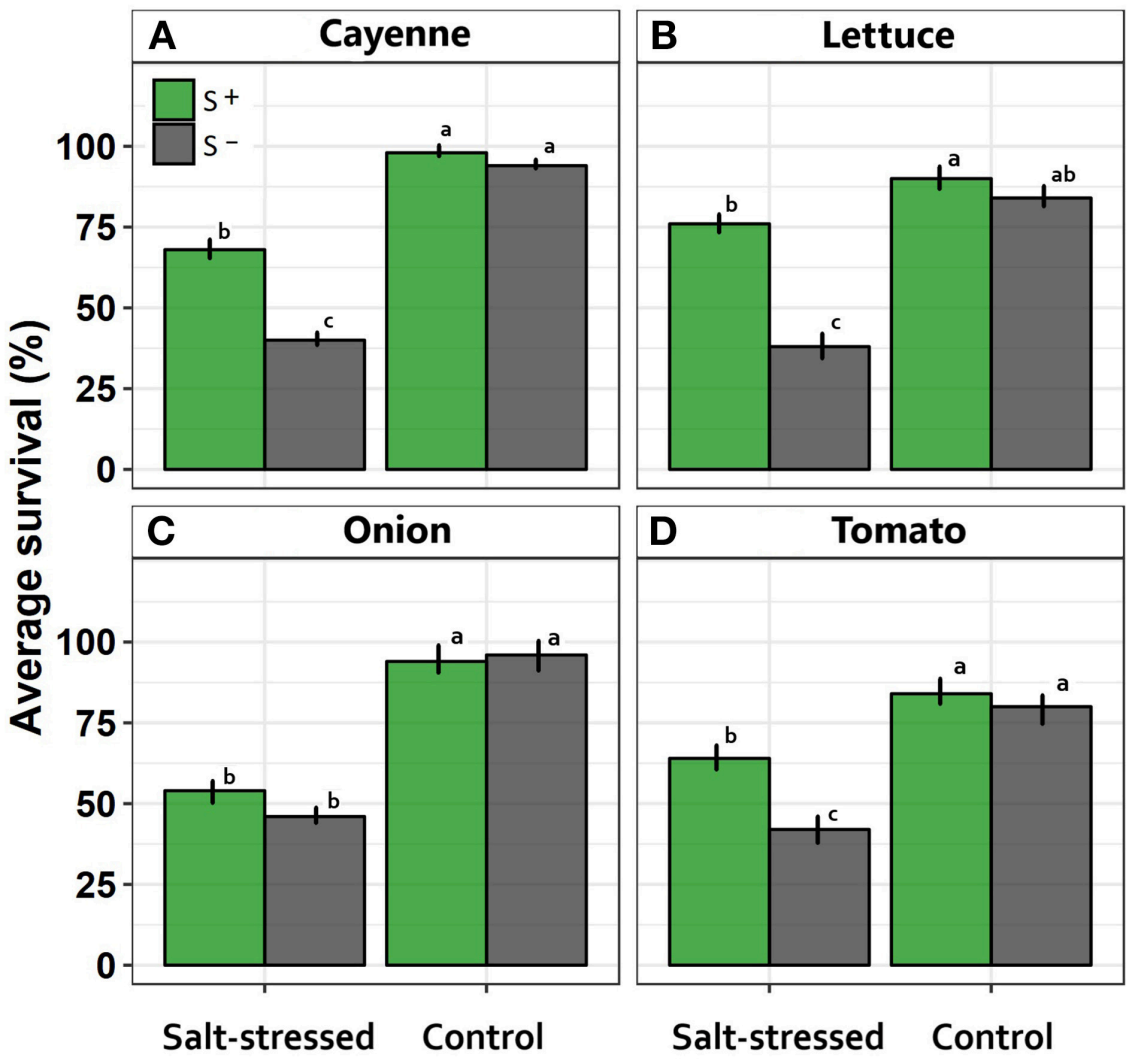
Effects of saline treatment (control vs. salt-stressed) and inoculation with a mix of Antarctic microorganisms (S– vs. S+ plants) on survival probability along time, in **(A)** cayenne, **(B)** lettuce, **(C)** onion, and **(D)** tomato crops. Different lowercase letters indicate significant differences between treatments (Tukey HSD tests; α = 0.05) independently for each species. Error bars represent ± SE values.

Average survival was greater in all crop species assessed under control compared with salt-stressed treatment (Figure 1). Similarly, symbiont consortium increase the survival in the majority of crops, with the exception of onion where survival percentage in salt-stress treatment was not different between inoculated vs. uninoculated individuals (Figure 1).

Specifically, symbiont consortium significantly increased the survival percentage in cayenne crop [*F*_(1, 16)_ = 30.12; *p* = 0.004; Figure 1A]. Contrarily, salt treatment significantly decreased the survival percentage in cayenne crop [*F*_(1, 16)_ = 207.53; *p* < 0.0001]. Interaction between inoculation x salt was significant [*F*_(1, 16)_ = 16.94; *p* = 0.008] since salt treatment decreased the survival with greater intensity in the treatment without symbiont consortium (Figure 1A). In the same way, interaction between inoculation x salt in lettuce crop was significant [*F*_(1, 16)_ = 28.44; *p* = 0.0067] because the survival average decrease under salt condition, being more evident in uninoculated condition (Figure 1B). On the other hand, onion crop showed a significant decrease in survival under salt-stress condition [*F*_(1, 16)_ = 337.50; *p* < 0.0001], but was not different among inoculated vs. uninoculated condition [*F*_(1, 16)_ = 4.17; *p* = 0.238; Figure 1C]. Finally, tomato crop showed a significative interaction between inoculation vs. salt [*F*_(1, 16)_ = 9.01; *p* = 0.0083] since those uninoculated individuals showed a more evident decrease in the survival percentage under salt-stress treatment (Figure 1D).

### Effects of the Saline-Stress

**Figure 2:**
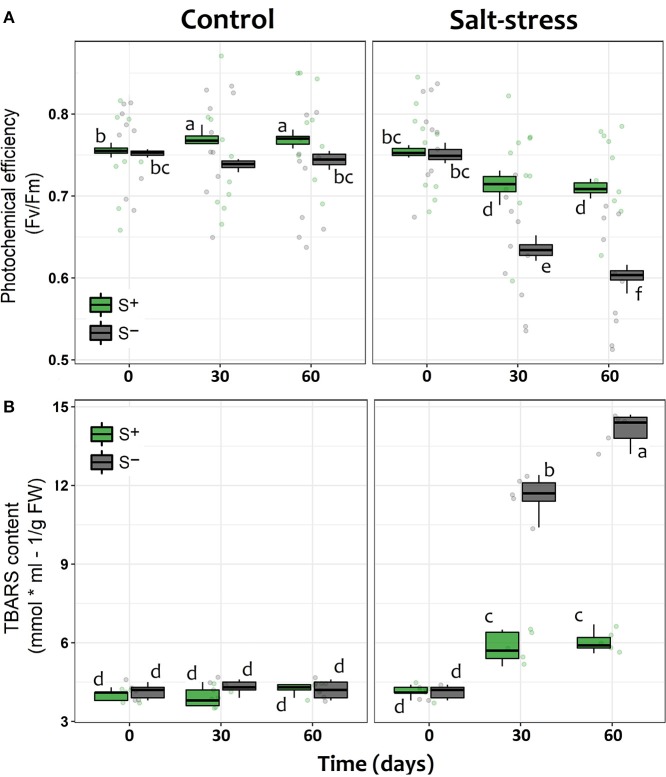
Physiological performance of lettuce individuals measured as **(A)** the individual photochemical efficiency (F_v_ /F_m_) of photosystem II (PSII) and **(B)** the level of lipid cell membrane peroxidation (TBARS) measured in leaves at 0, 30, and 60 days after exposed to salt stress (200 mM NaCl for “stressed” individuals) and inoculated with the Antarctic microbial consortium (S+ plants). Control groups for both conditions (non-stressed and non-inoculated, S–) are also showed. The box-plot represents the interquartile distribution of the data for each experimental group. Different letters indicate significant *a posteriori* differences (Tukey test, α < 0.05).

Photochemical efficiency, measured as F_v_/F_m_, showed the expected trend in plants under saline stress in both S– and S+ individuals, since their photosynthetic performance along time appeared to be negatively affected compared with control individuals without salt addition (Table 1). However, as it could be seen in stressed individuals after 30 and 60 days of saline treatment, the presence of the microbial symbionts significantly ameliorated the negative impact of saline stress. Symbiont appeared to stabilize the decrease of F_v_/F_m_ with time (Figure 2A). By contrast, among non-inoculated individuals under salt stress, the values of F_v_/F_m_ showed a continuous decrease along time, being the F_v_/F_m_ value at end of the experiment significantly lower than those recorded at day 30 (Figure 2A). Interestingly, the symbiotic interaction significantly enhanced the F_v_/F_m_ values of lettuce plants, even if they were not under saline stress; S+ individuals where already 3.8% more efficient in their photochemical performance than S– plants by the 30th day, and this difference was maintained in time (Figure 2A). On the other hand, the amount of TBARS showed a substantial increase for both S+ and S– plants under saline stress. Nevertheless, among S+ individuals, this increase was less than a half of what was observed for their S– counterparts (Figure 2B). Conversely, TBARS concentrations showed no differences at any time in plants without saline stress, despite their symbiotic status (Figure 2B).

**Table 1:** Repeated measures ANOVA for the effect of saline stress (S) and microbial symbiosis (Sy) on two variables of physiological performance (**a**: photosynthetic efficiency; **b**: lipid peroxidation) and two specific stress-response mechanisms (**c**: proline concentration; **d**: *NHX1* relative expression).

**Response variable**	**Source of variation**	***df*_**num**_**	***df*_**den**_**	***F***	***p***
Photochemical efficiency (Fv/Fm)	Stress (S)	1	72	908445.8	<0.0001
	Symbiont (Sy)	1	36	1226.8	<0.0001
	Time (T)	1	36	547.8	<0.0001
	Stress × Symbiont	2	72	366.3	<0.0001
	Stress × Time	1	36	149.1	<0.0001
	Symbiont × Time	2	72	396.4	<0.0001
	S × Sy × T	2	72	165.6	<0.0001
Lipid peroxidation (TBARS [MDA])	Stress (S)	1	32	16724.88	<0.0001
	Symbiont (Sy)	1	16	816.535	<0.0001
	Time (T)	1	16	382.987	<0.0001
	Stress × Symbiont	2	32	487.53	<0.0001
	Stress × Time	1	16	330.013	<0.0001
	Symbiont × Time	2	32	452.058	<0.0001
	S × Sy × T	2	32	207.78	<0.0001
Proline concentration (mmol/g FW)	Stress (S)	1	16	901.146	<0.0001
	Symbiont (Sy)	1	16	137.093	<0.0001
	Time (T)	2	32	185.671	<0.0001
	Stress × Symbiont	1	16	117.031	<0.0001
	Stress × Time	2	32	171.692	<0.0001
	Symbiont × Time	2	32	30.934	<0.0001
	S × Sy × T	2	32	29.946	<0.0001
*NHX1* gene expression (Relative fold change)	Stress (S)	1	32	513.41	<0.0001
	Symbiont (Sy)	1	16	90.69	<0.0001
	Time (T)	1	16	1065.19	<0.0001
	Stress × Symbiont	2	3.2	23.23	0.0002
	Stress × Time	1	16	755.79	<0.0001
	Symbiont × Time	2	32	92.31	<0.0001
	S × Sy × T	2	32	31.47	<0.0001

*The table shows the probability value (p) obtained for the significance of each factor in the fitted linear mixed model, which was obtained by maximizing the restricted log-likelihood for the data*.

### Mechanisms of Saline-Stress Tolerance

**Figure 3:**
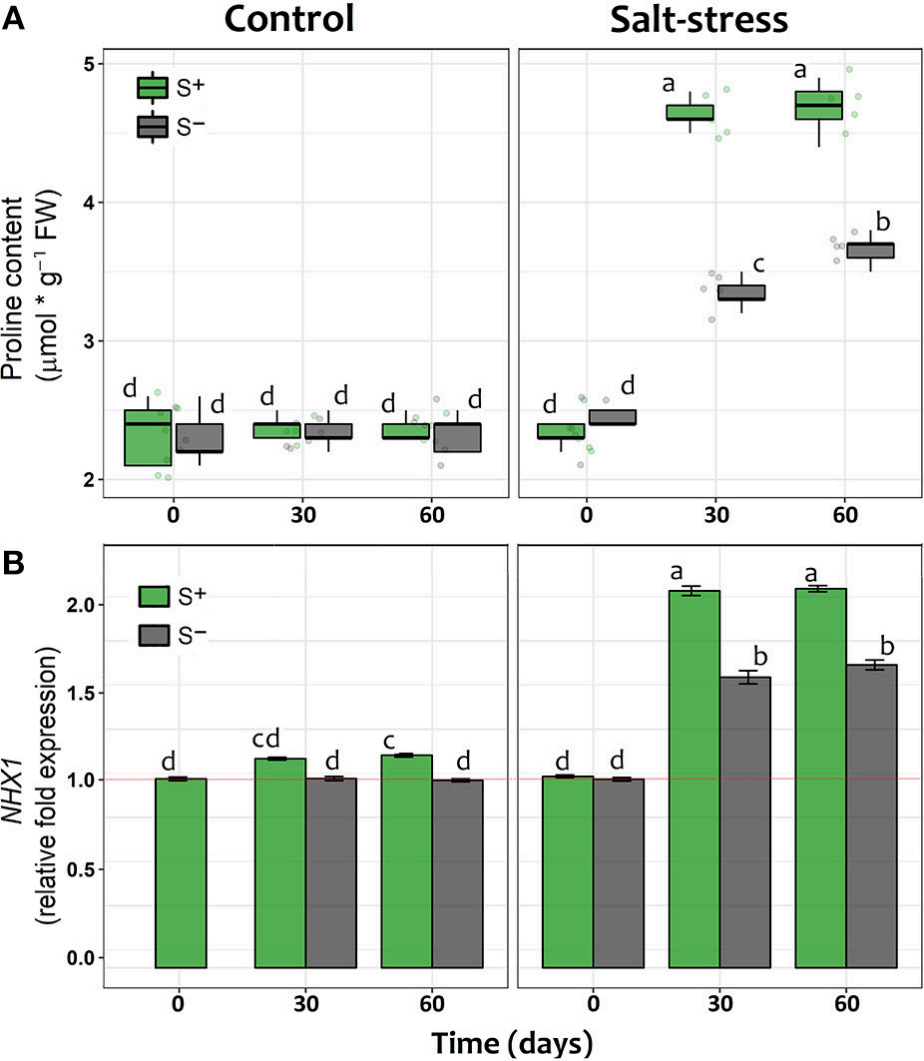
**(A, B)** Proline concentration (mmol/g FW) and, relative fold gene expression of *NHX1* in shoot tissue of lettuce plants measured at 0, 30, and 60 days after exposed to salt stress (200 mM NaCl for “stressed” individuals) and inoculated with the Antarctic microbial consortium (S+ plants). Control groups for both conditions (non-stressed and non-inoculated, S–) are also showed. The relative expression increases are referred to the mean levels of expression in control lettuce plants (i.e., non-stressed and non-inoculated) at time 0, indicated by a red line. The box-plot represents the interquartile distribution of the data for each experimental group. Error bars at the *NHX1* plot represent ± SE values. Different letters indicate significant a posteriori differences (Tukey test, α < 0.05).

The concentration of proline significantly increased after 30 and 60 days in salt-stressed individuals compared to non-stressed plants (Figure 3A). However, whilst S– individuals under saline stress showed an increase in proline concentration of 32% (day 30) and 48% (day 60), plants inoculated with the antarctic symbiotic consortium this increase achieved a 96 and 98%, respectively, during the same period (Figure 3A). As expected, among individuals without salt addition the mean proline concentrations were observed to be independent of time and symbiotic interaction (Table 1). In addition, described as a genetic response to saline-stress (Khan et al., 2013), the relative expression of *NHX1* showed larger changes in time among salt-stressed individuals compared with non-stressed S– and S+ plants. Furthermore, under saline stress, major increases appeared to be related with the presence of the symbiotic consortium; at day 30 its increase with respect to the reference value (S– day 0) was 32% for S– but 59% for S+ plants, a difference that was maintained at day 60 (Figure 3B). Interestingly, the symbiotic interaction also changes the *NHX1* relative expression under non-stressed conditions, although small (9%), a significant increase was observed at day 30 among S+ plants. By contrast, the *NHX1* expression of S– individuals do not present changes along time (Figure 3B).

### Symbiont Consortium on Crops Productivity

**Figure 4:**
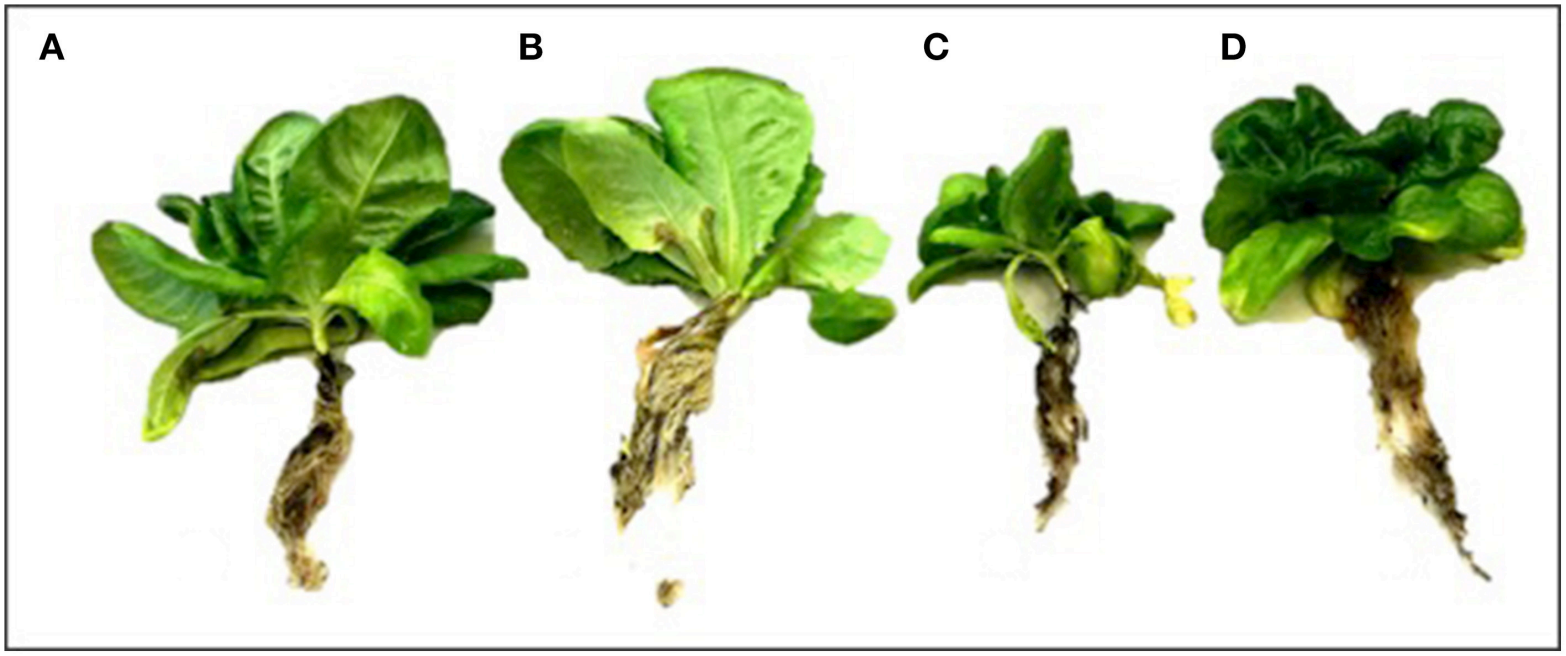
Morphology of lettuce plants after 90 day in each treatment. Uninoculated (S–) plant without salt stress **(A)**; Plants inoculated with a mix of Antarctic microorganisms (S+) not subjected to salt stress **(B)**; S– plants under salt stress (200 mM NaCl) **(C)**; S+ plant under salt stress (200 mM NaCl) **(D)**. Image credit: H. Hansen.

**Figure 5:**
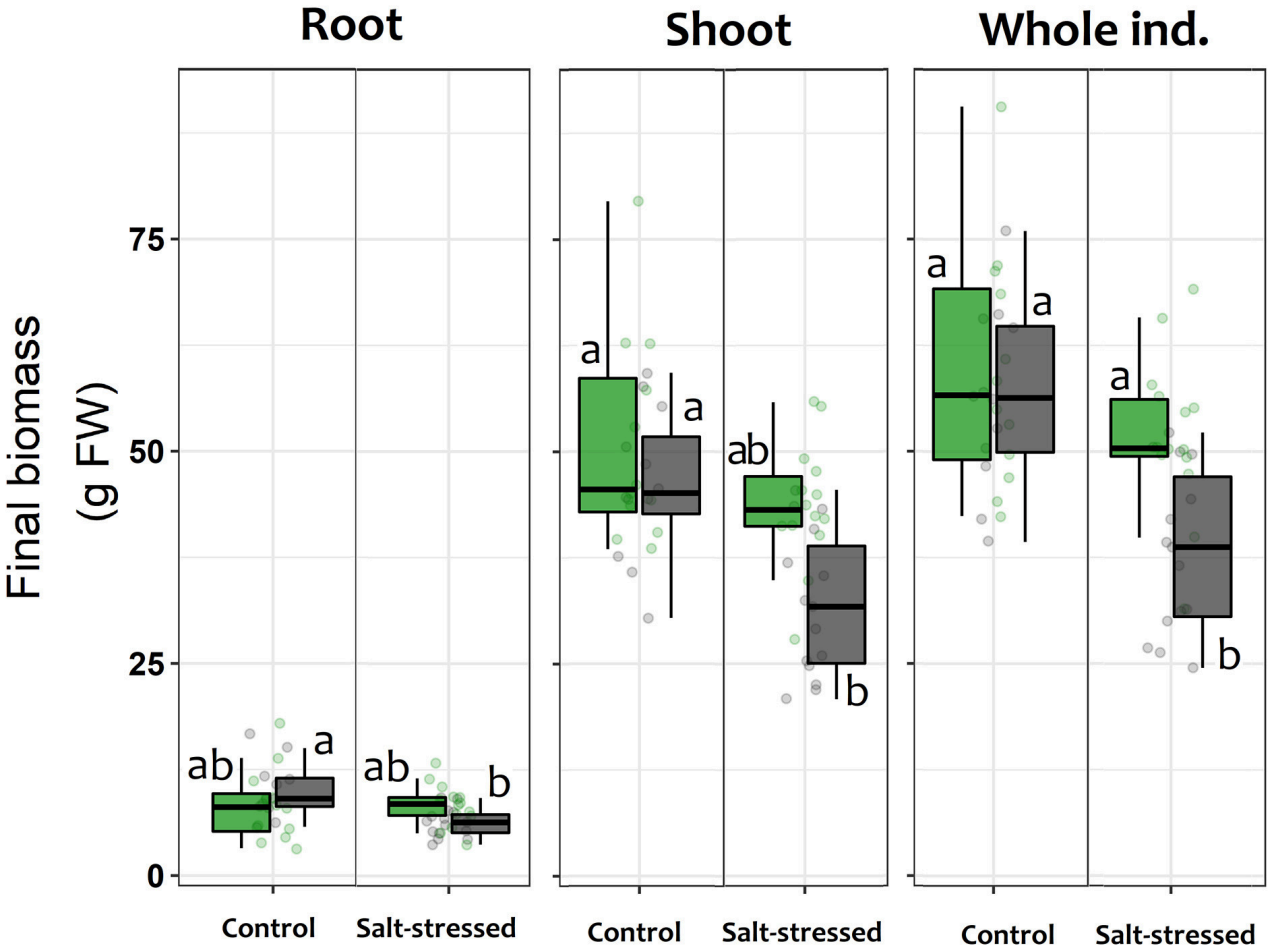
Final mean fresh biomass weight for roots, shoots and whole individuals of lettuce plants after being subjected for 60 days to saline stress (200 mM NaCl for “stressed” individuals) and inoculated with the Antarctic microbial consortium (S+ plants). Control groups for both conditions (non-stressed and non-inoculated, S–) are also shown. The box-plot represents the interquartile distribution of the data for each experimental group. Different letters indicate significant *a posteriori* differences (Tukey test, α < 0.05).

Overall, those lettuce individuals exposed to salt-stress treatment showed a smaller size than those allocated to control treatment or even those exposed to salt-stress but inoculated with the symbiont consortium (Figure 4). The two-way ANOVA on the final fresh biomass values showed a general negative impact of the saline growth conditions on productivity (factor salt-stress: *df* = 1.49; *F* = 5.502; *p* = 0.023, Figure 3); however, the significance of the ANOVA interaction term (i.e., inoculation x salt-stress: *df* = 1.49; *F* = 4.981; *p* = 0.031) suggests that this negative effect depends on the symbiotic status. In this sense, the root biomass reduction was only noticed among S– individuals, as could be observed on the a posteriori HSD-Tukey results (Figure 5). In lettuce plants under salt stress, the absence of microorganisms resulted in a decrease of 38% in the fresh biomass weight of roots respect to non-stressed plants. The same trend that was observed on the aboveground biomass, with a 31% mean shoot decrease among S– plants under saline stress and, consequently, on the whole plants (Figure 5). Interestingly, the two-way ANOVA for shoot tissues alone and for the whole individuals, despite shown significant *p*-values for “inoculation” and “salt-stress” as separate terms, do not presented a significant interaction in term of “inoculation x salt-stress” (shoot: *df* = 1.49; *F* = 1.777; *p* = 0.188; entire plant: *df* = 1,49; *F* = 3.047; *p* = 0.0871). This suggests a role of both factors in the observed results, however, with an apparent independence between them. Despite this, a-posteriori significant differences on either biomass variables could only be observed between stressed and non-stresses S– plants (Figure 5). By contrast, between both, salt-stressed and non-stressed S+ lettuce plants, we did not find any statistical differences between mean fresh weight of neither root or shoot tissues, nor for the whole plant.

## Discussion

In our study we showed evidence suggesting the importance of symbiont microorganisms from Antarctica improving salt tolerance in crops. In fact, under salt-stress condition the symbiont consortium significantly enhanced the survival in three of four crops assessed here. Hence, selected microorganisms from Antarctic plants could be a successful tool to avoid the negative effect of salt stress to others plant species as crops.

Beyond confirming that Antarctic environments are a fructiferous source of microbial extremophiles with potential applications, the results obtained in this work also unveil part of the mechanisms by which these symbiotic partners could benefit their host-plants. Compared with non-inoculated individuals, an enhanced proline accumulation and increased expression of the *NHX1* gene was observed among inoculated lettuces when they were subjected to salt-stress. Additionally, plants that were carrying the symbiotic consortium showed lower levels of cellular damage by lipid peroxidation and higher photosynthetic efficiencies.

Regarding the concentration of osmolytes, as a reported stres S–tolerance mechanism (e.g., glutamate, threalose, peptides, and other N-acetylated amino acids), proline appears to be particularly relevant to reduce the stressful effects of salinity (Hayat et al., 2012; López-Gómez et al., 2014; Reddy et al., 2015; Forni et al., 2017). The derived benefits of proline accumulation for a plant exposed to adverse environmental conditions (i.e., saline soils), could be related with the prevention of electrolyte leakage (Shahid et al., 2014), maintenance of the cell turgor (Ben Ahmed et al., 2011), and/or oxidative burst prevention (Hayat et al., 2012). In our work, the content of proline increased in both inoculated and non-inoculated individuals under salt stress. However, its accumulation in the later appeared to be slower, suggesting a role of the inoculated microorganisms in this process. In this sense, despite it is not possible with our design to differentiate the effects of the fungal and bacterial components of the microbial consortium, the proline-related response observed among inoculated lettuce plants may be originated by rhizobacterial symbionts, as other authors have shown (Kohler et al., 2009; Kumari et al., 2015). According to Creus et al. (2004), the plant-PGPR interaction frequently involves the control of the plant–water dynamics, facilitating the consequent enhanced potential for osmotic adjustment. Thus, it is not surprising that PGPR inoculation has been reported to result in enhanced tolerance to salt stress in several crops (Glick et al., 1997; Mayak et al., 2004; Yildirim and Taylor, 2005; Barassi et al., 2006; Egamberdieva et al., 2013; Forni et al., 2017).

Root-fungal endophytes could also have profound effects on plant performance as they can impact several components of their fitness; for example, by facilitating the uptake of essential nutrients like Potassium, Nitrogen or Phosphorus (Barka et al., 2002; Tanaka et al., 2005). This may have a direct impact in the physiological processes of the plant that are N-limited like carbon assimilation, and ultimately, growth, especially in soils with low degradation rates of organic matter in the rhizosphere (Jumpponen and Trappe, 1998; Upson et al., 2009). In this regard, diverse enzymes from root-fungal endophytes have been observed to degrade organic molecules like cellulose, lipids, starch, and complex proteins (Petrini et al., 1993; Caldwell et al., 2000; Suryanarayanan et al., 2012). For this reason, it has been proposed that the metabolic activity of these endophytes propitiates the nutritional enhancement of the rhizosphere in which their plant-host is developing (Newsham, 2011).

More similar to the proline response, the augmentation of the *NHX1* gene expression observed among inoculated individuals represents a direct effect of the interaction on the response capacity of the host-plant against saline stress. Linked with the synthesis of the NHX antiporter proteins, the expression of the *NHX1* gene has been associated with pH control and Na^+^/ K^+^ homeostasis (Leidi et al., 2010; Bassil and Blumwald, 2014), cell expansion (Apse et al., 2003) and salt tolerance (Hernández et al., 2009; Bassil and Blumwald, 2014). A generally accepted mode of operation of this protein is the transport of either K^+^ or Na^+^ into vacuoles in exchange of an H^+^efflux to the cytosol (Bassil and Blumwald, 2014). These antiporters also contribute to K^+^ uptake which is stored in specific vacuoles for turgor-generation and cell pH regulation (Leidi et al., 2010). This prevents toxic Na^+^: K^+^ ratios in the cytosol while accruing solutes for osmotic balance (McCubbin et al., 2014), for which the vacuolar accumulation of these elements is an especially crucial feature for plants under osmotic stress (Jiang et al., 2010).

For example, the PGPR *B. subtilis* can also decrease the absorption of excessive amounts of Na^+^ by the roots of plants by down-regulating the expression of the high affinity K^+^ transporter (*HKT1*) in the roots of salt-affected plants (Zhang et al., 2008; Qin et al., 2016). In addition, the effect of *B. subtilis* also was related with the shoot-to-root Na^+^ recirculation by triggering the induction of *HKT1* in shoots (Zhang et al., 2008). On the other hand, inoculation with fungal endohytes like *Piriformospora indica* have also resulted in increases in the expression of *NHX1* in salt-grown *Arabidopsis* compared to non-inoculated individuals (Abdelaziz et al., 2017). In our study, inoculated lettuce plants induced high levels of *NHX1* transcripts both under control and salt-stress conditions. However, it is under saline conditions where the relative expression increase appeared to be relevant, reaching almost a two-fold increase in the expression compared with non-inoculated plants.

In conclusion, we could determine that the selected consortium of Antarctic extremophiles effectively reduces the physiological impact of saline stress in a salt-susceptible crop like lettuce, as observed at both the individual and cellular levels. Interestingly, in terms of photochemical efficiency, the studied symbiosis leads to increases in plant performance even in the absence of saline stress. Nevertheless, under saline-stress, the responses of inoculated lettuce plants largely exceed those of non-inoculated individuals, both in terms of proline concentration and *NHX1* relative expression. Furthermore, as a consequence of this enhanced saline-stress tolerance, inoculated plants finalize their productivity cycle with a higher size than their non-inoculated counterparts. This may respond to the higher levels of photosynthetic efficiency sustained over time even under saline conditions, complemented with the decrease in the oxidative effects derived from saline-related ions, which appeared to be controlled by great amounts of NHX antiporters. The role of proline in stabilizing the cellular membranes under salt stress was also evident from the reduced lipid peroxidation measured as TBARS in inoculated plants, which showed almost a three-fold reduction compared with non-inoculated but stressed individuals.

Finally, the use of microorganisms associated with plants from stressful sites appears as a powerful tool for improving salt stress tolerance in intolerant (susceptible) crops. A variety of symbiotic microorganisms are now being used worldwide with the aim of enhancing plant productivity, especially on agricultural crops under saline stress conditions (Kim et al., 2014; Kaushal and Wani, 2016; Orhan, 2016; Qin et al., 2016; Singh and Jha, 2016; Etesami and Beattie, 2018). However, while most studies analyzed the effect of a single symbiotic partner, the high diversity of occurring microorganisms also implies a wide spectrum of physiological and biochemical effects. In this sense, while it is expected that we should aim to fully understand the nature of each plant-microbe relation, broad benefits for a potential plant-host could arise from the “consortium” approach to its symbiotic interactions (Sturz and Nowak, 2000; Faust, 2019).

## Author Contributions

MM-M, IA-R, and HH designed the experiments. MM-M, JG-C, and HH performed the experiments. MM-M, IA-R, HH, and CA analyzed the data. IA-R and MM-M wrote the paper along with HH, JG-C, and CA. All authors reviewed the manuscript.

### Conflict of Interest Statement

The authors declare that the research was conducted in the absence of any commercial or financial relationships that could be construed as a potential conflict of interest.
